# Periacetabular osteotomy with or without arthroscopic management in patients with hip dysplasia: study protocol for a multicenter randomized controlled trial

**DOI:** 10.1186/s13063-020-04592-9

**Published:** 2020-08-18

**Authors:** Geoffrey P. Wilkin, Stéphane Poitras, John Clohisy, Etienne Belzile, Ira Zaltz, George Grammatopoulos, Gerd Melkus, Kawan Rakhra, Tim Ramsay, Kednapa Thavorn, Paul E. Beaulé

**Affiliations:** 1 Division of Orthopaedic Surgery, The Ottawa Hospital, General Campus, 501 Smyth Road, Ottawa, ON K1H 8L6 Canada; 2 School of Rehabilitation, University of Ottawa, 451 Smyth Road, Ottawa, ON Canada; 3 Division of Orthopaedic Surgery, Washington University School of Medicine, 660 S Euclid Ave, St. Louis, MO USA; 4 Division of Orthopaedic Surgery, Centre hospitalier de l’Université Laval, Québec, QC Canada; 5 Division of Orthopaedic Surgery, William Beaumont Hospital (Troy Michigan), Royal Oak, MI USA; 6 Department of Clinical Epidemiology, Ottawa Hospital Research Institute, 501 Smyth Road, Ottawa, ON Canada

**Keywords:** Periacetabular osteotomy, Arthroscopy, Randomized control trial, Quality of life

## Abstract

**Background:**

Hip dysplasia is one of the most common causes of hip arthritis. Its incidence is estimated to be between 3.6 and 12.8% (Canadian Institute for Health Information, Hip and knee replacements in Canada, 2017–2018: Canadian joint replacement registry annual report, 2019; Jacobsen and Sonne-Holm, Rheumatology 44:211–8, 2004). The Periacetabular Osteotomy (PAO) has been used successfully for over 30 years (Gosvig et al., J Bone Joint Surg Am 92:1162–9, 2010), but some patients continue to exhibit symptoms post-surgery (Wyles et al., Clin Orthop Relat Res 475:336–50, 2017). A hip arthroscopy, performed using a small camera, allows surgeons to address torn cartilage inside the hip joint. Although both procedures are considered standard of care treatment options, it is unknown whether the addition of hip arthroscopy improves patient outcomes compared to a PAO alone. To delay or prevent future joint replacement surgeries, joint preservation surgery is recommended for eligible patients. While previous studies found an added cost to perform hip arthroscopies, the cost-effectiveness to Canadian Health care system is not known.

**Methods:**

Patients randomized to the experimental group will undergo central compartment hip arthroscopy prior to completion of the PAO. Patients randomized to the control group will undergo isolated PAO. Patient-reported quality of life will be the primary outcome used for comparison between the two treatment groups as measured by The International Hip Outcome Tool (iHOT-33) (Saberi Hosnijeh et al., Arthritis Rheum 69:86–93, 2017). Secondary outcomes will include the four-square step test and sit-to-stand (validated in patients with pre-arthritic hip pain) and hip-specific symptoms and impairment using the HOOS; global health assessment will be compared using the PROMIS Global 10 Score; health status will be assessed using the EQ-5D-5L and EQ VAS questionnaires (Ganz et al., Clin Orthop Relat Res 466:264–72, 2008) pre- and post-operatively. In addition, operative time, hospital length of stay, adverse events, and health services utilization will be collected. A sub-group of patients (26 in each group) will receive a T1rho MRI before and after surgery to study changes in cartilage quality over time. A cost-utility analysis will be performed to compare costs and quality-adjusted life years (QALYs) associated with the intervention.

**Discussion:**

We hypothesize that (1) concomitant hip arthroscopy at the time of PAO to address central compartment pathology will result in clinically important improvements in patient-reported outcome measures (PROMs) versus PAO alone, that (2) additional costs associated with hip arthroscopy will be offset by greater clinical improvements in this group, and that (3) combined hip arthroscopy and PAO will prove to be a cost-effective procedure.

**Trial registration:**

ClinicalTrials.gov NCT03481010. Registered on 6 March 2020. Protocol version: version 3.

## Background

### Problem to be addressed

In the fiscal years 2017–2018, 58,492 hip replacements were performed in Canada, which reflects a 17.4% increase over the preceding 5 years [1]. More than $1 billion is currently spent annually performing joint replacement surgeries as a result of secondary osteoarthritis (OA) [1]. The prevalence of hip dysplasia in the general population ranges from 4.0 to 12.8% [2, 3]. Research shows that patients with hip dysplasia are 5 times more likely to develop OA than patients with normal hip morphology, eventually requiring a hip replacement [4]. More broadly, hip dysplasia has a long history of association with OA [5] and is considered the leading precursor to OA across North America [6–8] and Europe [9, 10]. Wylie and Peters have established a clear relationship between the severity of deformity (as per radiological findings) and risk for OA development [5]. The more shallow acetabulum results in a smaller surface area of cartilage for weight bearing and leads to overload of the acetabular rim and labrum [11]. This leads to progressive damage of the cartilage in the hip joint and eventually causes arthritic changes [6], as demonstrated by Kim et al.’s correlation between lack of proteoglycan content and severity of arthritic symptoms [12]. Prior to the development of advanced OA, patients often have a prolonged phase of painful hip symptoms that interfere with activity and quality of life [13, 14]. These symptoms usually affect younger adult patients, who will require a hip replacement surgery if not treated for their dysplasia [13]. Fortunately, hip dysplasia can be diagnosed before more severe damage occurs and treated through hip preservation surgery (including periacetabular osteotomy) [6].

The Bernese periacetabular osteotomy (PAO) reorients the more shallow and vertically oriented acetabulum and has been used successfully for over 30 years as the standard of care for hip dysplasia [15]. Garbuz et al. showed that hip dysplasia significantly affects quality of life and that surgical correction with PAO leads to a significant improvement [14]. In addition, PAO has been shown to limit radiographic changes in 88% and 75% of hips at 10- and 20-year follow-ups respectively [16, 17]. The optimization of joint surface area in the weight-bearing region of the hip and improvements in the mechanical advantage of the abductor musculature are thought to provide most of the symptomatic improvement post-operatively. PAO outcomes have shown good symptomatic improvements, low complication rates, and an 18-year hip joint survival of 74% [18], whereas patients with untreated hip dysplasia have shown a 20-year hip joint survival of 33% [4]. More recently, Wyles et al. showed that for patients undergoing a PAO for symptomatic dysplasia, the rate and pattern of progression approximated those for patients with normal morphology from the historical cohort as well as significantly improved when compared with patients with unmanaged DDH. Similarly, in patients with Tönnis grade 1, the probability of progression to total hip arthroplasty at 5 and 10 years was 2% and 11%, respectively, compared with 23% and 53%.

A PAO is not only recommended to *delay or prevent* the need for joint replacement surgery later on in life [10, 19], but *positively impact the quality of life* in patients with hip dysplasia. By doing a PAO, we can offset the need for a future hip replacement, which also saves money, as a PAO has been demonstrated as a more cost-effective procedure than a total hip replacement [20].

Despite the breadth and depth of evidence highlighting the benefits following a PAO, 60–85% of patients have concomitant intraarticular pathology (cartilage damage) that cannot be corrected with a PAO [21, 22] and 11% continue to exhibit symptoms post-surgery [23]. Patients with hip dysplasia commonly have concomitant labral tears [24–26], which has been suggested to be responsible for residual symptoms after PAO [27]. Although MRIs have been routinely used to identify intraarticular pathology, they have been shown to be unreliable to appropriately detect them and quantify their severity [28]. A hip arthroscopy, performed using a small camera, allows surgeons to address the intraarticular pathology inside the hip joint. As a result, some have advocated combining arthroscopic labral re-fixation/debridement and PAO [29–31]. Between 2006 and 2010, there has been a 600% increase in hip arthroscopies in the USA [32], with similar trends in Canada, demonstrating its high uptake since the mid-2000s. Although hip arthroscopy allows the surgeon to potentially address other intraarticular pathologies (i.e., ligamentum teres, chondral injuries), the effect of arthroscopic management of these injuries concomitantly with PAO is unknown. Furthermore, it is unknown whether cartilage damage can be mitigated with the addition of an arthroscopic procedure [33]. Due to additional operative time, equipment, and human resources, costs are substantially increased by adding an arthroscopy to a PAO [34, 35]. In Ontario, the addition of hip arthroscopy to the PAO represents a 125% increase in cost just in the OR [36]. While the cost-effectiveness of a PAO has been established in the US healthcare (cost-effectiveness of $7856 per quality-adjusted life year and an average incremental effectiveness of 0.15) [20], a true cost-utility benefit must be demonstrated if arthroscopic management of labral and other intraarticular pathologies can be justified in these patients.

## Methods/design

### Principal research questions

The primary objective of this project is to assess the impact of concomitant hip arthroscopy with PAO for patients with symptomatic hip dysplasia on quality of life and cartilage health when compared to patients undergoing PAO only. The secondary objective is to investigate the cost utility of performing arthroscopy with a PAO from a healthcare utilization and societal perspective.

Our hypothesis is that concomitant hip arthroscopy at the time of PAO to address intraarticular pathology will result in significant improvements in quality of life and prevent further cartilage damage when compared to PAO alone. Also, we hypothesize that the additional costs associated with hip arthroscopy will be offset by greater clinical improvements and decreased cost consequences in this group and that combined hip arthroscopy and PAO will prove to be a cost-effective procedure.

### The need for a trial

Dysplasia of the hip is one of the main causes of secondary OA [6–10]. Literature has demonstrated that PAO effectively alters the progression of hip dysplasia [19]. Over the past 3 years, there have been three systematic reviews looking at the clinical impact of arthroscopy for hip dysplasia, with all concluding potential benefits of the procedure from pre-experimental studies, but highlighting the absence of randomized controlled trials limiting the utility of the results [37–39]. Furthermore, there have been two systematic reviews looking at the contribution of arthroscopy, PAO, or a combination of both procedures in patients with hip dysplasia, both concluding the lack of robust studies concerning the combination of the two procedures [40, 41].

According to the 2019 systematic review [39], there have been four studies looking at the clinical benefit of addressing labral and/or other intraarticular pathology at the time of PAO for hip dysplasia. All of these studies have been non-randomized cohorts [22, 30, 42, 43], so the true magnitude of clinical benefit from the combined interventions has been impossible to determine. Three of these studies saw clinical benefits when adding arthroscopy to PAO, while the fourth did not. The last study involved an additional intervention (osteochondroplasty), rendering the isolation of arthroscopic labral refixation/debridement as a possible benefit difficult. In addition to their pre-experimental design, the other three studies demonstrating positive results also possess serious biases limiting the validity of the results, such as low sample sizes, inequivalent groups at baseline, patient allocation bias, and lack of standardization of co-interventions. Thus, the role labral refixation/debridement played in the eventual outcome in addition to PAO remains uncertain.

Additionally, no study to date has conducted a cost-effectiveness analysis comparing the two surgical treatment strategies. Two studies were recently conducted in the USA comparing the cost-effectiveness of hip arthroscopy compared to non-operative management of labral tears in young adults [44, 45], while both studies found that although there is an added cost to perform hip arthroscopies, it was more cost-effective compared to non-operative management. Furthermore, no study to date has compared the cost-effectiveness of periacetabular osteotomy with and without hip arthroscopy, in relation to patient quality of life. As hip arthroscopy is a relatively new procedure and even more so as adjunct to the PAO, it is critical to demonstrate its benefits to patient outcome as well as cartilage health.

### Proposed trial design

The proposed trial design follows the same design as our completed feasibility pilot trial, to which we began recruiting in January 2019. Logistical and protocol issues have been addressed and resolved through close collaboration between the clinical research coordinators and surgeons to ensure a streamlined approach. As such, we are set up to efficiently and effectively conduct a multisite randomized, parallel group superiority trial with two sites in Canada (The Ottawa Hospital and Centre Hospitalier Universitaire de Québec) and two sites in the USA (William Beaumont Hospital in Detroit and Washington University in St Louis).

### Planned trial interventions

All patients will undergo a Bernese periacetabular osteotomy (PAO) as the standard of care for treatment of symptomatic hip dysplasia/hip instability. The PAO will be completed by the treating surgeon’s usual technique using a rectus femoris-sparing approach. All surgeons have over 10 years of experience with both techniques of hip arthroscopy and PAO as stand-alone procedures and thus are past the learning curve for both procedures. After completion of the PAO, the patient’s range of motion will be assessed intra-operatively. Once optimal correction is achieved with PAO, a range of motion of the hip is done, and if flexion less than 100° is achieved and/or less than 20° of internal rotation at 90° of flexion is achieved, the surgeon will relieve the impingement at either the sub-spine area, i.e., anterior inferior iliac spine, or the femoral head/neck junction through an anterior arthrotomy (Additional file 2).

All patients will receive standardized DVT prophylaxis and standardized heterotopic ossification prophylaxis with non-steroidal anti-inflammatory medications (NSAIDS) for a minimum of 10 days post-operatively unless medical contra-indications exist. Protected weight bearing on the operative leg will be applied for a minimum of 6 weeks post-operatively [46]. After this period, all patients will undergo a standardized exercise protocol to be accomplished at home following these guidelines [47]: standing open-chain active hip abduction and flexion and knee extension sitting. These exercises will be performed for 2 weeks. For the following 2 weeks, step up/step down and side-stepping (with resistance band) will be accomplished. All exercises will be performed every other day, with three sets of 10 repetitions. Patients will be asked to complete an exercise diary to assess and promote adherence (Additional file 3). Patients will be instructed to contact their treating surgeon if they experience prolonged pain or discomfort after exercise.

Physical examination and radiographic follow-up assessments and measurements will be performed at regular intervals until the PAO has completely healed as part as standard of care. Radiographic control (well-centered AP pelvis and 45° Dunn view) done at the pre-operative time point, 2–4 weeks, 6 months, 12 months, and 24 months post-operatively, is the current standard of care at all institutions involved in the study [48]. Additional x-rays may be performed at the surgeon’s discretion. In addition to the standard of care treatment described above, patients will be randomized to either the *experimental group* or the *control group.*

Patients randomized to the *experimental group* will undergo central compartment hip arthroscopy prior to completion of the PAO. Diagnostic arthroscopy will first be performed, and intra-operative findings will be recorded on a standardized reporting form (Additional file 4). Hip arthroscopy will be carried out using the treating surgeon’s usual technique and will adhere to the following guidelines: a limited inter-portal capsulotomy may be made to allow sufficient space for visualization and instrumentation, if needed (a “T” capsulotomy should not be performed); labral refixation using a minimum of two suture anchors for any identified unstable labral tears; labral debridement should be reserved for only clearly irreparable tears or very small, stable tears; maximal preservation of any remaining intact labral tissue should be attempted; treatment of chondral lesions and ligamentum teres pathology may be performed at the surgeon’s discretion; and arthroscopic cam decompression should not be performed (Additional file 2). Patients randomized to the *control group* will undergo isolated PAO.

### Randomization

Randomization will be carried out in blocks randomly varying between 2 and 4 to ensure equal numbers in both treatment arms (i.e., “PAO” and “Scope-PAO”). Randomization will be stratified by recruiting site to allow for even distribution of intervention covariates and patient characteristics across each site and by sex to ensure adequate distribution of this variable for the analyses. Patients will be randomized just after enrolment, because MRI sensitivity is inadequate to identify intraarticular pathologies [28]. This will allow the surgeon’s office and treating institution to appropriately plan for the required surgical intervention, necessary equipment, operating room time, etc. Patients will be randomized through an on-line computer randomization program that will be accessible by designated research staff from all participating sites.

### Proposed methods for protecting against sources of bias

The addition of hip arthroscopy represents a discrete intervention added on to the current standard of care for treatment of symptomatic hip dysplasia (i.e., PAO). Thus, a randomized trial represents the best study design for assessing its potential additive effect. Since the treating surgeon must administer the experimental intervention, a double-blind design is not possible.

Assessments that are not patient self-report and data analyses will be performed blinded to allocation. When submitting our feasibility protocol to our Research Ethics Board in the spring of 2018, we initially had indicated blinding of study participants through a diagnostic arthroscopy in the PAO-only patients (same incision as PAO+arthroscopy group). However, there were ethical implications with inserting a scope into the hip of the control group and therefore we converted the study design to a single-blinded methodology only, recognizing that this may introduce a potential source of bias in any patient-reported outcome measures. In addition to patient-reported outcomes, objective performance measurements, administered by blinded assessors, will be performed as one of the outcome measures. At each postoperative time point, patients will document through a standardized form conservative and surgical co-interventions targeting the treated hip and received after the previous time point.

### Inclusion criteria

- Skeletally mature patient undergoing Bernese PAO for symptomatic acetabular dysplasia/hip instability, as defined by Wilkin et al.’s validated Ottawa Classification for Symptomatic Acetabular Dysplasia [49]
- Patients having over 6 months of non-surgical management including physical therapy and NSAIDs
- Between 16 and 50 years old
- Patient capable of giving informed consent to participate

### Exclusion criteria

- Pregnancy
- Prior hip/pelvis surgery of any kind on the surgical side
- Prior hip arthroplasty surgery on either side
- Concurrent proximal femoral osteotomy and/or surgical hip dislocation
- Radiographic evidence of arthritis (i.e., Tönnis grade ≥ 2)
- Systemic inflammatory disease
- Known connective tissue disorder (e.g., Ehlers-Danlos syndrome)
- Known neuromuscular disorder (e.g., cerebral palsy, spina bifida)
- Known skeletal dysplasia (e.g., achondroplasia, multiple epiphyseal dysplasia)
- Cognitive impairment (individuals who, based on the treating surgeon’s assessment during the pre-operative discussion, are incapable of providing informed consent for treatment. In other words, if the patient requires a substitute decision maker to consent to surgery on their behalf, they would be excluded from study participation)
- Patient unable/unwilling to complete all required follow-up visits
- Patient unwilling to participate

### Primary outcome measure

#### iHOT-33

Disease-specific patient-reported quality of life will be the primary outcome used for comparison between the two treatment groups as measured by the International Hip Outcome Tool (iHOT-33). The iHOT-33 is a reliable, validated, self-administered quality of life assessment tool for young, active patients with hip symptoms [50]. The iHOT-33 is a core measure for this patient population [51, 52]. Patients will complete this questionnaire pre-operatively and at the following time intervals post-operatively: 6 months, 12 months (primary time point), and 24 months (Additional file 5). The value of the iHOT-33 at each time point will be used for analysis. Data will be aggregated such that the mean scores in each treatment group will be compared at each time point. In addition, the proportion of patients in each treatment group that have an increase in their score greater the minimum clinically important difference (MCID) of the score will be compared.

### Secondary outcome measures

#### Performance measures

Physical performance measures will be assessed, which are less susceptible to bias due to patient unblinding. The four-square step test and sit-to-stand five times test will be used, as they have been validated in patients with pre-arthritic hip pain with effect sizes of − .63 and − .61, respectively [53]. These assessments will be completed at each site preoperatively, at 6 months, 12 months, and 24 months postoperatively. The score on these tests at each time point will be used for analysis. Mean scores between treatment groups will be compared at each time point.

#### PROMIS Global 10

General health-related quality of life will be measured using the PROMIS Global 10 assessment (Additional file 6). This 10-item tool assesses general domains of health-related quality of life in the domains of physical, mental, and social well-being [54]. Patients will complete this questionnaire pre-operatively and at the following time intervals post-operatively: 6, 12, and 24 months. The score at each time point will be used for analysis. Mean scores between treatment groups will be compared at each time point.

#### MRI T1ρ cartilage mapping

The association between hip arthroscopy outcomes and cartilage quality is not well elucidated in the literature. The quality of the hip articular cartilage will be assessed by a non-invasive and validated MRI technique called T1ρ mapping that enables the evaluation of proteoglycan content [55, 56]. The cartilage will be segmented on co-registered anatomical MR images and quantitative cartilage T1ρ values will be measured in six regions over the cartilage [57]. The T1ρ relaxation time is sensitive to the cartilage proteoglycan content and increasing when the proteoglycan content is decreasing. We will compare the cartilage T1ρ relaxation times at the initial scan and at the follow-up scan (24 months after surgery) in the different regions to investigate changes in the cartilage macromolecule content after surgery. The significance of the differences in T1ρ will be tested using a paired t-test with a significance level of *p* < 0.05.

#### Adverse events

Adverse events will be systematically queried and documented prospectively for the first 90 days [58] using Sink et al.’s classification system that grades the complications based on required treatment and long-term morbidity [59] (Additional file 7). Any other unexpected adverse events that are reported by the study participants will also be recorded for the duration of the study. The type of adverse event will be narratively reported in any publications along with their severity using the classification of Sink et al. [58]. Reoperation rate will also be monitored at 12 and 24 months post-operatively in each group using standardized failure modes [60] (Additional file 7). Although a recent retrospective chart review has demonstrated that there were no differences in reoperation rates between PAO alone and PAO combined with arthroscopy at minimum 2-year follow-up [61], there are obvious limitations in regard to selection bias, a prospective randomized trial might show otherwise (see Figure 4 in Additional file 8 for the figure as per Spirit guidelines).

### Health economic measures

A cost-utility analysis will be performed to compare costs and quality-adjusted life years (QALYs) associated with the intervention and the standard of care over a 24-month period. The analysis will be conducted from the perspective of Canada’s publicly funded healthcare system, and resource use will be measured via a retrospective chart review. Only Canadian sites will be included in the economic analysis because of the important differences in healthcare systems between countries, with general consensus among health economists that power issues do not apply to these types of analyses. We will include the intervention costs (i.e., PAO and arthroscopy procedure) and health utilization-related costs. Health utility scores will be derived from the PROMIS score for each trial participant using a validated algorithm [62]. QALYs will be estimated using the total area under the curve method [63]. The statistical analysis will be conducted in accordance with current guidelines for clinical and cost-effectiveness analysis alongside RCTs [64]. The incremental cost and QALY will be estimated using generalized estimating equations that explicitly allows for the modeling of normal and non-normal distributional forms of repeated measure data. The cost per QALY gained will be obtained through the difference in average costs of the two treatment options divided by the difference in average QALYs as denoted by the coefficient of the therapy indicator variables. Uncertainty in the analysis will be addressed by estimating 95% confidence intervals using a non-parametric bootstrapping method. As a secondary analysis, we will use a societal perspective and consider productivity loss as measured through the work productivity and activity impairment questionnaire (WPAI:SHP) (completed at 6, 12, and 24 months), which has been used in studying patients after elective orthopedic surgery [65] (Additional file 9).

### Sample size and power calculation

A total sample size of 126 subjects is planned. Calculation of the required sample size is based on ensuring sufficient statistical power to detect a difference in the primary outcome measure (i.e., iHOT-33) at 12 months. The standard deviation of the iHOT-33 is approximately 16 in pre-arthritic patients [66–68]. The original iHOT-33 manuscript reported a minimal clinically important difference (MCID) of 6.1 in a general symptomatic hip population [66]. Since then, three other studies focusing on hip arthroscopy reported MCIDs varying from 10 to 12 [67–69] in time ranges varying from 6 to 12 months. Using a non-central *t* function with a conservative MCID of 10, an alpha level of 0.05, a power of 0.90, and a loss to follow-up of 15% provides a total sample size of 63 per group (total of 126).

For the T1ρ analysis, part of the subjects from each of the two subgroups will undergo quantitative hip cartilage MRI T1ρ-mapping. Our group has shown T1ρ to be a valid alternative to the gold standard (dGemric) for patients with symptomatic hip dysplasia (Additional file 1). Based on our hypothesis that patients undergoing in arthroscopy+PAO will conserve cartilage characteristics, whereas those with PAO only will demonstrate affected cartilage, the sample size for this study is calculated using a power analysis on T1ρ data from our longitudinal cartilage T1ρ study (two time points, mean follow-up time of 24.5 months) on corrective surgery for cam deformity in association with FAI [70]. Two years after surgery, T1ρ decreased significantly (*p* < 0.05) in the anterosuperior area of cartilage from 34.6 to 29.4 ms reaching control T1ρ values. By assuming a standard deviation of the mean T1ρ value of 5%, a power of 0.9, and alpha error of 0.05, we calculated that 21 subjects are needed for each of the two subgroups. Based on the follow-up loss rates from the former study (5 of 24; 20%) with this patient population, we will recruit a total of 26 patients per subgroup which will undergo the additional cartilage T1ρ-mapping protocol [57].

### Analyses and frequency of analyses

The analysis will be conducted on an intention-to-treat basis. Statistical analyses will be performed blinded to treatment allocation. A level of significance of 0.05 will be used for all analyses. The underlying assumptions of all statistical procedures will be evaluated, and data transformation will be considered. Using descriptive statistics, baseline patient characteristic differences between the study groups will be assessed (age, sex, outcomes) and differences will be controlled for in the statistical analyses. Repeated measures mixed models and general linear mixed models will be used for handling continuous and dichotomous outcomes respectively. Sex will be specifically assessed as an effect modifier in the analyses. Multiple imputation will be considered as a sensitivity analysis.

For cartilage health, the biochemical content of the cartilage will be evaluated using MRI T1ρ mapping. The scan will be performed pre- and 2 years after surgery using a 3D turbo spin echo acquisition method with T1ρ preparation (CUBE QUANT) and the following parameters: TR = 1350 ms, TEeff = 40 ms, FOV = 25.6 × 25.6 cm^2^, in-plane resolution = 0.5 × 0.5 mm^2^, slice thickness = 3 mm, number of slices = 32 slices, no slice gap (3D sequence), B1 = 500 Hz, spin-lock times = 1, 15, 30, 45 ms, and scan time = 18 min [56, 71] (Additional file 1, Figure 3). For post-pressing, T1ρ-weighted datasets will be co-registered to the proton density-weighted turbo spin echo images and analyses described in our previous publications [57, 70]. The hip cartilage will be divided in six regions of interest (ROIs): anterior lateral, anterior middle, anterior medial, posterior lateral, posterior middle, and posterior lateral for regional proteoglycan assessment [57]. T1ρ values from the different cartilage regions will be compared and evaluated for significant differences between the pre-operative and the follow-up scan using repeated measures analysis of variance (ANOVA).

In addition to the final analysis, an interim analysis will be performed after 2.5 years to determine if the study must be terminated. This will be based on the following results: serious adverse events significantly higher in the experimental group, greater than expected beneficial effects, improbable statistically significant difference by the end of the study, and participant recruitment is severely lagging and unlikely to achieve the target.

### Confidentiality and retention of records

To maintain confidentiality of participant data, a data management plan will be developed and approved by the coordinating center. Study participant medical information obtained by this study is confidential, and disclosure to third parties other than those noted in the Informed Consent Document is prohibited. All local and federal guidelines and regulations regarding maintaining study participant confidentiality of data will be adhered to. Data generated by this study must be available for inspection by representatives of the Coordinating Center or their representative, Study Monitoring personnel, and the IRBs.

Each participating center is responsible for tracking all of their enrolled patients and storing paper records according to local IRB guidelines. The master study database containing participant information will be stored in a password-protected excel file stored in the secure, in-house hospital server at the coordinating center. The password will only be known to members of the approved study team.

Participants will not be identifiable in any publications or presentations resulting from this RCT study. No identifying information will leave the participating center. All information which leaves the participating centers will be coded with an independent study number.

Research records will be retained in accordance with site IRB policies. The starting date used to calculate the retention period is the date when a record is first created. All paper records will be stored in a securely locked office by the participating center study coordinators. Upon receiving data from the participating centers, all electronic records will be stored on the OHRI secure server which is physically and virtually protected.

### Secondary use of data

Any secondary use of data for a purpose other than what is listed in the study protocol will require additional ethics approval. If the new purpose is to obtain data not yet collected from participants, it will require re-consenting of all existing study participants.

#### Protocol amendments

Should any amendments or modifications to the study protocol be necessary during the course of the study, a protocol amendment submission will be made to the REB of the lead site. Once approved, the details of the protocol amendment will be communicated directly to the designated study coordinators at each participating site via email. If the amendments will have any effect on the patients already enrolled in the study, they will be notified by telephone by the study coordinator at each site. Any trial registries or journals that have published the protocol will also be notified of the amendments via the agency’s approved notification mechanism.

#### Post-trial care

All participants will continue to receive medical care from their treating surgeon after trial completion, as necessary. The are no provisions for providing compensation for patients who suffer harm from participation in the trial.

#### Authorship

Authorship for any trial publications will be based the authorship guidelines of the publication. In general, authors must have materially contributed to any or all of the following study aspects: protocol design, treatment provision, data analysis, manuscript preparation, and critical review. There is no intention to use professional writers.

## Discussion

### Planned recruitment rate, organization, and time period

As aforementioned, we have already obtained Research Ethics Board approval for our feasibility trial. An amendment will be submitted at the lead site and Centre Hospitalier Universitaire de Québec, to add the T1ρ component. We estimate that approval of this amendment will take 2 to 3 months at both sites. Since January 2019, we have recruited 36 patients overall across four sites as part of the feasibility cohort (on average 6 patients per month). Based on similar studies completed by this multidisciplinary study team, an average recruitment rate of 8–10 patients per month is more than feasible as the recruitment strategy is optimized with proper human resources [72, 73].

Enrolling patients from four centers will allow sufficient numbers in an adequate timeframe to achieve power in our statistical analyses. Multiple participating centers/surgeons will also improve the applicability of the results and translation of the findings to various surgeon practices and patient populations. An average of 60 PAOs are performed at each US site and approximately 40 PAOs are performed at each Canadian site per year, thus providing approximately 200 potentially eligible patients for recruitment per year. Including Washington University in St. Louis Missouri and the William Beaumont Hospital in Detroit Michigan will allow us to achieve timely recruitment rates, due to their higher volume. Including Centre Hospitalier Universitaire de Québec will allow a broader Canadian perspective. Involving sites across North America will allow for stronger generalizability of study findings. We expect a comparable consent rate for the proposed randomized control trial as for the feasibility pilot at 72 patients per year. Taking into account an eligibility rate of 75% and a consent rate of 60%, recruitment will take 1.5 years to complete (Additional file 10), with 126 patients needed to achieve sufficient statistical power.

Subjects will be recruited prospectively from the surgical practices of the participating surgeons at the participating sites. All patients that meet the inclusion criteria (above) will be considered for enrollment. The total number of patients considered for enrollment will be recorded, whether or not they are eventually included in the research study. For patients that meet the inclusion/exclusion criteria, the surgeon will provide a brief description of the study as well as a relevant discussion of the surgical risks and benefits of hip arthroscopy and periacetabular osteotomy. Patients that express interest in participation will then be contacted by the surgeon’s Clinical Research Coordinator who will re-confirm eligibility and provide a detailed description of the study and obtain written informed consent for participation (see Informed Consent Form used for pilot study in additional attachments). Once patient consent to participate in the study has been obtained, the patient will complete a surgical consent form with the procedure listed as “[Left/Right] periacetabular osteotomy, +/− hip arthroscopy.” Participation is voluntary and patients may withdraw consent for participation at any time.

Patients recruited in the T1ρ analysis will be recruited from the above cohort at The Ottawa Hospital and at the Centre Hospitalier Universitaire de Québec over the span of one and a half years. The patient will be invited to be additionally assessed with T1ρ. This will leave 4 years to complete the collection of all primary and secondary outcome measures, to reach the primary endpoint of 2 years. The cost-utility analysis will occur in years 4 and 5 (see Additional file 10).

### Anticipated compliance problems

We do not anticipate compliance problems, as the interventions are one-time surgeries. If patients cross over from one treatment group to another after randomization, their results will still be included in the final analysis on an intention-to-treat basis. All attempts should be made to avoid patient cross-over and if cross-overs do occur after randomization, the reason for this will be recorded. The aim of the pilot was to test the methods and optimize data collection procedures. If that data is shown to be applicable to the current proposal, it will be included.

### Anticipated loss to follow-up rate

Compliance with all follow-up assessments will be ensured by the Clinical Research Coordinator and each participating site and may include contacting patients by phone and/or email to ensure compliance with follow-up assessments. If patients withdraw from participation prior to surgery, they will not be included in the final analysis. If patients withdraw from participation after surgery, the outcome scores completed prior to their withdrawal will be included in the final analysis. It is anticipated the loss of follow-up will be around 15%, with around 20% for imaging at 2 years. This is based on loss to follow-up rates seen in similar trials with similar follow-up periods [41, 70]. Randomized controlled trials accomplished at our site have also typically seen 15% loss to follow-up rates.

### Multidisciplinary team

The proposed multidisciplinary team of surgeons and researchers has the necessary breadth and depth of clinical and research experience to carry out this study. As demonstrated through successful recruitment of 36 patients into the feasibility trial, Drs. Beaulé, Wilkin, Zaltz, Belzile, and Clohisy have the patient volume and resources to recruit their patients. In addition, as part of the ANCHOR group, these centers have been engaged in the study of large prospective patient cohorts of young adults with hip pain publishing over 30 peer-reviewed papers. With close collaboration, the protocol has been refined to allow for optimal standardization across all sites. The outstanding experience and broad range of disciplines will ensure successful completion of the trial.

### Day to day management of trial

As Nominated Principal Applicant, Dr. Paul Beaulé will lead this trial and supervise the team of surgeons and researchers. The Clinical Research Coordinator at each of the participating sites will perform data collection and ensure accurate recording and storage of collected data. All data will be kept secure and all records will be retained for the prescribed duration after trial completion in accordance with site-specific policies. All data will be compiled for analysis at the lead site.

### Trial steering committee and independent data safety monitoring board

A trial steering committee will be instituted every 6 months to review study safety and accrual. The committee will convene every 6 months and will be composed of the nominated principal applicant, principal applicants, co-applicants, research coordinators, and patient representative at the lead site. Recruitment, data quality, adherence, and safety issues will be monitored. An independent data safety monitoring board (DSMB) will include surgeons and a biostatistician not involved in the trial who will compare by intervention groups baseline variables, primary and secondary variables, safety or adverse event variables, and adherence measures for the entire group. The DSMB will meet 1 year after the start of recruitment, and then every 6 months onwards. The DSMB will be responsible for the interim analysis.

### Using the results of this trial

This project is particularly innovative since it is the first randomized controlled trial to study the clinical outcomes and cost utility of concomitant arthroscopy and PAO. The addition of a cost-effectiveness analysis is also particularly relevant in the current environment of increasing demands on the healthcare system within a single-payer system and the need to improve cost-efficiency of healthcare delivery. It will also be the first study to assess the value for money of the two surgical treatment strategies worldwide. Furthermore, no study to date has assessed the impact of concomitant arthroscopy with labral treatment and PAO on cartilage health. There have been several studies using advanced magnetic resonance imaging (MRI) for cartilage before and after PAO, showing its effect on the cartilage matrix [33], but none yet with the adjunct of hip arthroscopy. Hence, it is imperative to determine how the addition of labral repair/debridement may affect the hip cartilage health/status. Although most of the work on cartilage imaging in hip dysplasia has been done with dGEMRIC, it requires the use of IV gadolinium. Our work using the MR relaxation time (T1ρ) has been shown to be sensitive to the proteoglycan content and change in the hip joint without the need of a contrast agent [56] (Additional file 1, Figure 1. Our preliminary work comparing T1ρ to dGEMRIC in patients has shown significant correlation (Additional file 1, Figure 2). Consequently, adding quantitative T1ρ mapping assessing the hip cartilage condition pre- and post-operatively comparing hip arthroscopy vs. no hip arthroscopy in the setting of a PAO is innovative. This is especially relevant as it has been shown using patient specific finite element modeling [74] that although a PAO favorably alters the joint mechanics at the labral-chondral junction, joint congruency is decreased which could be further impacted if the labral structure is altered with the addition of hip arthroscopy.

The results from this study will be shared among the Academic Network of Conservational Hip Outcomes Research (ANCHOR) Collaborative Network. The ANCHOR network consists of a group of surgeons with a goal of improving the surgical care and quality of life for patients with pre-arthritic hip disorders. An abstract for the manuscript will be submitted for presentation at national orthopedic meetings in both Canada and the USA (i.e., the Canadian Orthopaedic Association and the American Academy of Orthopaedic Surgeons annual meetings). An abstract will also be submitted to international meetings such as the International Society for Hip Arthroscopy. There will be approximately 4 manuscripts as a result of this study published in high impact orthopedics journals (i.e., Journal of Bone and Joint Surgery). A policy brief will also be prepared and distributed to relevant policy makers. As our knowledge users, Dr. George Grammatopoulos (in Canada), Dr. Christopher Peters (in the USA), and Dr. Taryn Taylor (Sports Medicine Physician in Canada) will use the findings of this study to inform their high-volume hip preservation surgery practices and council their patients in regard to surgical options. Better understanding the outcomes following PAO with and without concomitant arthroscopy has the capacity to increase awareness at the provider level (Sports Medicine Physicians) and better highlight the importance of early intervention and prevention of a more costly procedure down the line (i.e., hip replacement surgery).

### Risks to safety of study participants

The risk of a PAO performed without hip arthroscopy may lead to untreated labral tears that may result in ongoing pain and require arthroscopy at a later time [60]. The risks of a PAO performed with a hip arthroscopy include the following: increased surgery time, fluid extravasation into the soft tissues resulting in increased hip/leg swelling, traction on the leg possibly increasing the risk of a nerve injury, partial capsulotomy which may lead to hip instability, and inadvertent damage to the labrum or cartilage as well as intra-articular adhesions. Although there are additional risks to performing an arthroscopy in addition to a PAO, it is hypothesized that arthroscopy will address intraarticular pathology, thereby minimizing cartilage damage and improving quality of life.

## Trial status

The first protocol (version 1) was dated 9 January 2018. Recruitment under that protocol, also known as the “feasibility trial,” began on 18 May 2018. The current feasibility study protocol version is 11 April 2019 (version 2), which is now completed. With the addition of performance measures based on this written protocol dated 06 March 2020 (version 3), we will re-start recruitment.

## Supplementary information

**Additional file 1: Figure 1.** Demonstrating sensitivity of T1rho to proteoglycan content and change in the hip joint without the need of a contrast agent. **Figure 2.** Spearman rank correlation coefficient (rho: − 0.60, *p* < 0.001*). **Figure 3.** Cartilage Mapping.

**Additional file 2.** Standardized Operative Procedures.

**Additional file 3.** Exercise Diary.

**Additional file 4.** PAO ± Hip Arthroscopy Study.

**Additional file 5.** International Hip Outcome Tool (iHOT-33).

**Additional file 6.** PROMIS Global Health 10.

**Additional file 7.**

**Additional file 8: Figure 4.** Schedule of enrolment, interventions, and assessments.

**Additional file 9.** Work Productivity and Activity Impairment Questionnaire: Specific Health Problem V2.0 (WPAI:SHP).

**Additional file 10: Table 2.** Study Gantt Chart.

## Data Availability

The study team members involved in the operations of this trial will be the only ones with access to the final trial dataset. Fully executed contracts between the Lead Site and participating sites are already in place to allow for data sharing for the final analyses, which will be performed by the Lead Site.
