# Supplementary material for: Periacetabular osteotomy with or without arthroscopic management in patients with hip dysplasia: study protocol for a multicenter randomized controlled trial
Source: Trials. 2020 Aug 18;21:725. doi: 10.1186/s13063-020-04592-9 (PMC7433104; doi:10.1186/s13063-020-04592-9)
Supplement: Supplementary file 1 — Additional file 1: Figure 1. Demonstrating sensitivity of T1rho to proteoglycan content and change in the hip joint without the need of a contrast agent. Figure 2. Spearman rank correlation coefficient (rho: − 0.60, p < 0.001*). Figure 3. Cartilage Mapping. [file 13063_2020_4592_MOESM1_ESM.pdf]

Appendix A

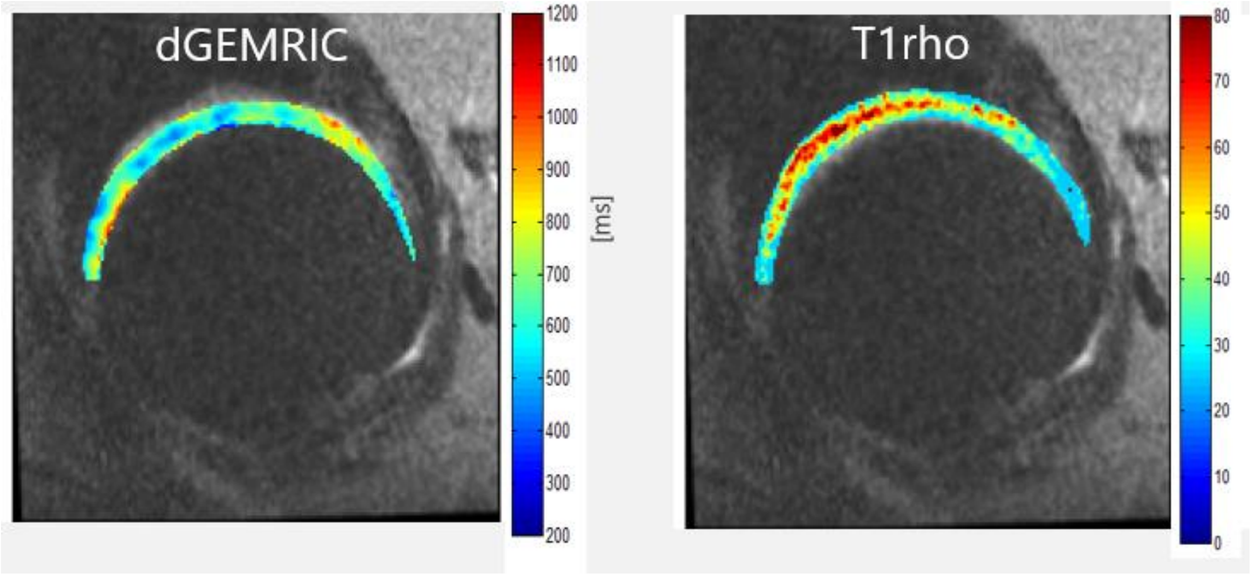

Figure 1. Demonstrating sensitivity of T1rho to proteoglycan content and change in the hip joint without the need of a contrast agent

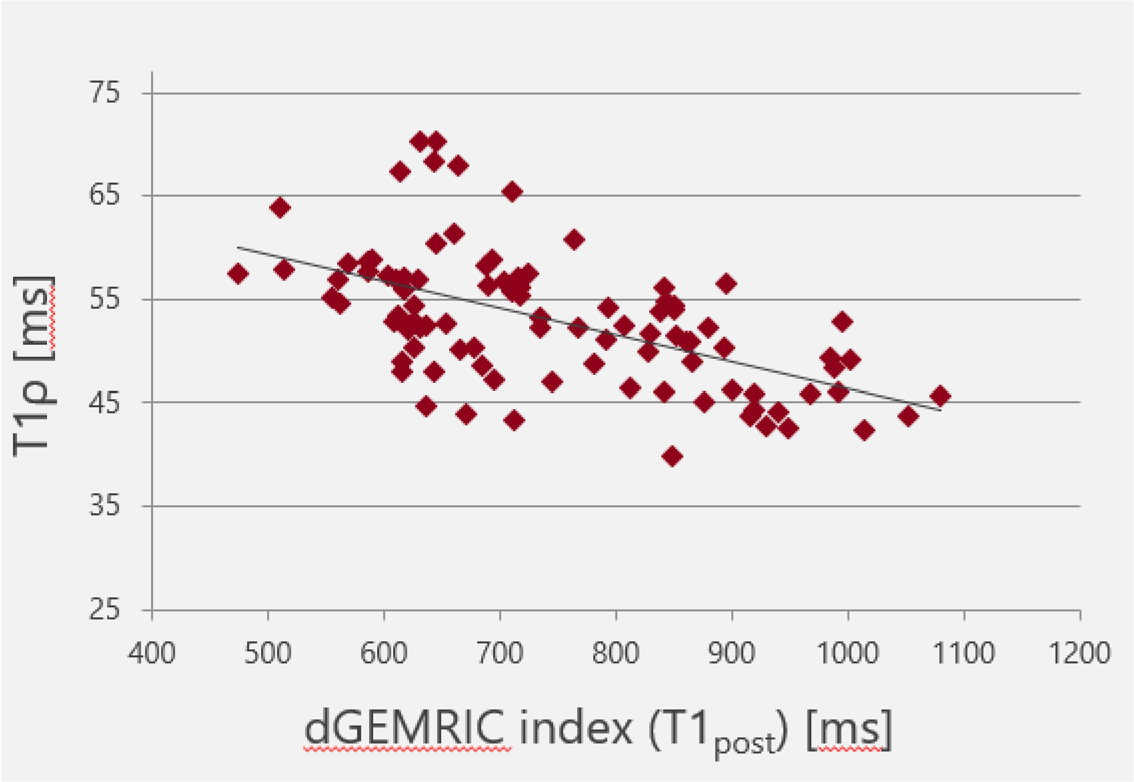

Figure 2. Spearman rank correlation coefficient (rho: -0.60, p<0.001*)

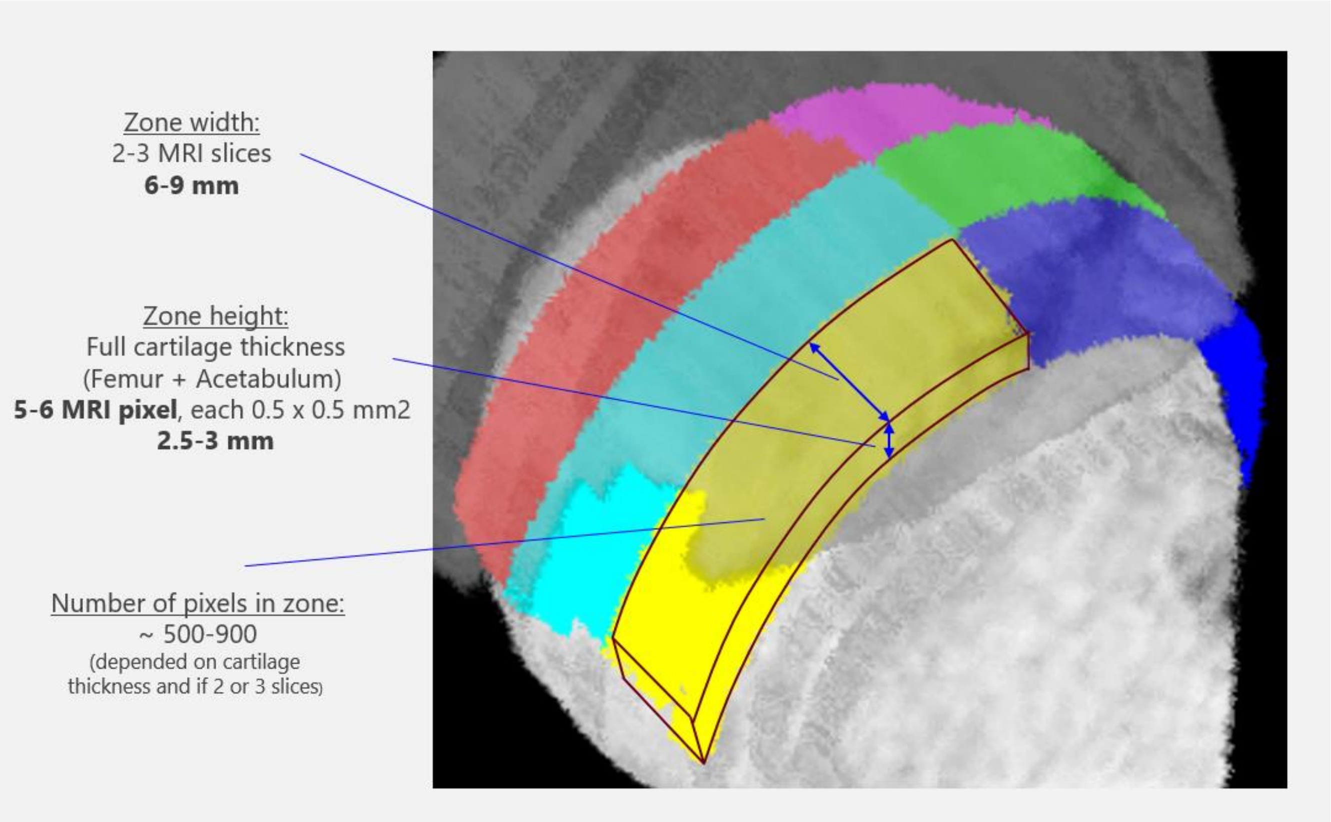

Figure 3. Cartilage Mapping
