# Supplementary material for: Periacetabular osteotomy with or without arthroscopic management in patients with hip dysplasia: study protocol for a multicenter randomized controlled trial
Source: Trials. 2020 Aug 18;21:725. doi: 10.1186/s13063-020-04592-9 (PMC7433104; doi:10.1186/s13063-020-04592-9)
Supplement: Supplementary file 2 — Additional file 2. Standardized Operative Procedures. [file 13063_2020_4592_MOESM2_ESM.pdf]

Appendix B
Standardized Operative Procedures

1. *Standardized hip arthroscopy.*
   - We have established consensus among ANCHOR surgeons on arthroscopic treatment of central compartment disease including indications for labral repair, debridement of ligament teres and routine utilization of capsular closure.
   - Modern labral treatment
     a. Labral repair – labrochondral detachment with adequate tissue quality for repair
     b. Labral debridement – poor labral tissue or labral ossification
   - Capsular closure a. Suture based closure of arthroscopic capsulotomy (2 or more sutures, closure of at least 75% of capsulotomy)

2. *Standardized Peri-acetabular osteotomy*
   - Modern PAO technique as described by Clohisy et al(1) will be done which has been established with consensus with the ANCHOR group.
   - Correction of fragment will be aimed with LCE angle of >25 degrees and less than 35 degrees with a roof angle less than 10 degrees
   - Version of the acetabular fragment will be done to balance anterior and posterior wall coverage i.e.retroversion index less than 20°(2)
   - A minimum of 2 screws will be placed for fixation
   - If after correction, internal rotation is less than 20 degrees at 90 degrees of flexion the surgeon will remove bony impingement by either subspine decompresion and/or femoral head/offset restoration(3).

3. Low-dose preoperative CT
   - Low-dose CTs are utilized as standard of care in preoperative planning of surgical treatment as established by consensus meeting (4).

References

1. Clohisy JC, Barrett SE, Gordon JE, Delgado ED, Schoenecker PE. Periacetabular Osteotomy for the Treatment of Severe Acetabular Dysplasia. J Bone Joint Surg. 2005;87A(2):254-9.
2. Diaz-Ledezma C, Novack T, Marin-Peña O, Parvizi J. The relevance of the radiological signs of acetabular retroversion among patients with femoroacetabular impingement. The bone & joint journal. 2013;95(7):893-9.
3. Nassif NA, Schoenecker PL, Thorsness R, Clohisy JC. Periacetabular osteotomy and combined femoral head-neck junction osteochondroplasty: a minimum two-year follow-up cohort study. JBJS. 2012;94(21):1959-66.
4. Su AW, Hillen TJ, Eutsler EP, Bedi A, Ross JR, Larson CM, et al. Low-dose computed tomography reduces radiation exposure by 90% compared with traditional computed tomography among patients undergoing hip-preservation surgery. Arthroscopy: The Journal of Arthroscopic & Related Surgery. 2019;35(5):1385-92.
