# Supplementary material for: Periacetabular osteotomy with or without arthroscopic management in patients with hip dysplasia: study protocol for a multicenter randomized controlled trial
Source: Trials. 2020 Aug 18;21:725. doi: 10.1186/s13063-020-04592-9 (PMC7433104; doi:10.1186/s13063-020-04592-9)
Supplement: Supplementary file 4 — Additional file 4. PAO ± Hip Arthroscopy Study. [file 13063_2020_4592_MOESM4_ESM.pdf]

PAO ± Hip Arthroscopy Study

Appendix D

Time interval: Pre-op

**Patient Information**		
1. Subject ID:	__________________________________	
2. Operative hip:	☐ Left	☐ Right
3. Sex:	☐ Male	☐ Female
4. Month/year of birth (mmm/yyyy):	__________________________________	

Patient History

1. Pain location:<br>(check all that apply)	☐ Anterior (groin)<br>☐ Anterior (thigh)<br>☐ Lateral<br>☐ Posterior (buttock)<br>☐ Other (specifiy) :	2. Pain chronicity:<br>(select one)	☐ < 6 months<br>☐ 6 months - 1 year<br>☐ 1 - 3 years<br>☐ 3 - 5 years<br>☐ > 5 years

Date (dd/mmm/yyyy): ___________________ Participant ID: _________________________

PAO ± Hip Arthroscopy Study

Time interval: ☐ Pre-op ☐ 6 months ☐ 12 months ☐ 24 months post-op

Physical Exam

1. Height : ___________ cm 2. Weight: ___________ kg

3. Range of motion

1) Left leg:	2) Right leg:
Start of flexion: ___________ °	Start of flexion: ___________ °
End of flexion : ___________ °	End of flexion : ___________ °
IR at 90° flexion : ___________ °	IR at 90° flexion : ___________ °
ER at 90 ° flexion : ___________ °	ER at 90 ° flexion : ___________ °
Abduction : ___________ °	Abduction : ___________ °
Adduction : ___________ °	Adduction : ___________ °
IRE: ___________ °	IRE: ___________ °
ERE : ___________ °	ERE : ___________ °

Indicate positive or negative for each	positive	negative
4. 1) Left hip:		
Anterior impingement sign	☐	☐
Anterior apprehension (groin)	☐	☐
Posterior impingement pain (buttock)	☐	☐
2) Right hip:		
Anterior impingement sign	☐	☐
Anterior apprehension (groin)	☐	☐
Posterior impingement pain (buttock)	☐	☐

Indicate left or right for each	left	right
5. Prone-Apprehension Relocation Test (PART):	☐	☐
6. FADDIR:	☐	☐

Date (dd/mmm/yyyy): ___________________ Participant ID: _________________________

PAO ± Hip Arthroscopy Study

Time interval: ☐ Pre-op ☐ 2-4 weeks ☐ 6 months ☐ 12 months ☐ 24 months post-op

Radiographic Finding Digital Measurements

1. Anteroposterior pelvis: ☐ No ☐ Yes

If yes, answer the following:

1) Patient is: ☐ Standing ☐ Supine

2) Date of X-ray (dd/mmm/yyyy): __________________________

3) Tönnis classification (select one of the following):

☐ Grade 0 (no signs of osteoarthritis)

☐ Grade 1 (increased sclerosis of the head nad acetabulum)

☐ Grade 2 (small cysts in the head or acetabulum, moderate joint space narrowing, moderate loss of head sphericity)

☐ Grade 3 (large cysts in head or acetabulum, severe joint space narrowing or obliteration, severe deformity of femoral head, evidence of necrosis)

4) Lateral center edge angle (Wiberg): __________ °

5) Acetabular inclination (Tönnis angle): __________ °

6) Alpha angle: __________ °

7) Cross over sign: ☐ No ☐ Yes

If yes, select crossover location below:

☐ Superior 1/3 of acetabulum

☐ Middle 1/3 of acetabulum

☐ Inferior 1/3 of acetabulum

8) Posterior wall sign: ☐ No ☐ Yes

9) Prominence of ischial spin (PRIS) sign: ☐ No ☐ Yes

10) Congruency classification (select one of the following):

☐ Excellent

☐ Good

☐ Fair

☐ Poor

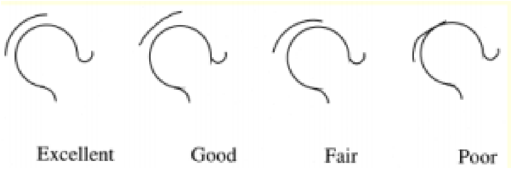

2. Faux profile view: ☐ No ☐ Yes

If yes, answer the following:

1) Date of X-ray (dd/mmm/yyyy): __________________________

2) Anterior roof angle: __________ °

3) Posterior joint space narrowing: ☐ No ☐ Yes

PAO ± Hip Arthroscopy Study

Radiographic Finding Digital Measurements (Continued)

3. Dunn view: ☐ No ☐ Yes

If yes, answer the following:

1) ☐ 45° or ☐ 90° Dunn

2) Date of X-ray (dd/mmm/yyyy): ___________________________

3) Alpha angle: __________ °

4. MRI study : ☐ No ☐ Yes

If yes, answer the following:

1) Date of MRI (dd/mmm/yyyy): ___________________________

2) Labral tear: ☐ No ☐ Yes

3) Acetabular cyst: ☐ No ☐ Yes

4) Femoral head cyst : ☐ No ☐ Yes

5) Alpha angle at 3 o’clock position: __________ °

6) Alpha angle at 130 position: __________ °

5. 3D CT scan ☐ No ☐ Yes

If yes, date of scan (dd/mmm/yyyy): ___________________________

PAO ± Hip Arthroscopy Study

Time interval: Intra-op

**Surgical procedure**

1. Arthroscopy: ☐ No ☐ Yes

If yes, select one of the following:

Beck cartilage damage

- ☐ 0 Normal (macroscopically sound cartilage)
- ☐ 1 Malacia (roughening of surface, fibrillation)
- ☐ 2 Pitting malacia (roughening, partially thinning and full-thickness defects or deep fissuring to the bone)
- ☐ 3 Debonding (loss of fixation to the subchondral bone, macroscopically sound cartilage; carpet phenomenon)
- ☐ 4 Cleavage (loss of fixation to the subchondral bone; frayed edges, thinning of the cartilage)
- ☐ 5 Defect (full-thickness defect)

☐ Labral damage

2. Concurrent procedures (select all that apply):

☐ Acetabular articular cartilage grafting	☐ Femoral head chondroplasty
☐ Acetabular articular cartilage fixation	☐ Femoral head microfracture
☐ Acetabular chondroplasty	☐ Femoral head/neck osteochondroplasty
☐ Acetabular microfracture	☐ Labral reconstruction (graft)
☐ Acetabular rim osteoplasty	☐ Labral recontouring/shrinkage/thermal stabilizatoin
☐ Adhesiolysis-head neck junction	☐ Labral refixation/repair
☐ Adhesiolysis-labrocapsular junction	☐ Labral resection (partial)
☐ Arthroscopic partial capsulectomy	☐ Labral resection (complete)
☐ Arthotomy	☐ Ligamentum teres debridement
☐ Arthroscopic capsular incision/closure (longitudinal)	☐ Ligamentum teres repair
☐ Capsular tightening	☐ Open arthrotomy
☐ Femoral head articular cartilage fixation	☐ Psoas lengthening/release
☐ Femoral head articular grafting	☐ Synovectomy
☐ Femoral head central resection	

3. Subspine (AIIS) decompression: ☐ No ☐ Yes

4. Duration of surgery from skin incision to wound closure: __________ min

5. Transexamic acid: ☐ No ☐ Yes

6. DVT prevention: ☐ No ☐ Yes

If yes, select from the following:

☐ Xarelto ☐ LMW Heparin ☐ ASA

☐ Other (specify: ____)

Date (dd/mmm/yyyy): __________________ Participant ID: ________________________

PAO ± Hip Arthroscopy Study

Time Interval: Track all adverse events within 90 day via retrospective chart review

Post-op Adverse Events

Please use the below grading scheme to indicate post-op adverse events.

Grade	Definition
**1**	**Any deviation from the normal postoperative course** without the need for pharmacological treatment or surgical, endoscopic and radiological interventions. Allowed therapeutic regimens are: drugs (antiemetics, antipyretics, analgesics, diuretics), electrolytes and physiotherapy.
**2**	**Requiring pharmacological treatment with drugs** other than those allowed for Grade 1 complications. Blood transfusions and total parenteral nutrition are also included.
**3a**	**Requiring surgical, endoscopic or radiological** intervention (not under general anaesthesia)
**3b**	**Requiring surgical, endoscopic or radiological intervention** (under general anaesthesia)
**4a**	**Life-threatening complication requiring ICU management** (single organ)
**4b**	**Life-threatening complication requiring ICU management** (multi organ)
**5**	**Death** of a patient

**Adverse events**	**No**	**Yes (select one)**						
		1	2	3a	3b	4a	4b	5
1. Airway/breathing	☐	☐	☐	☐	☐	☐	☐	☐
2. Cardiac arrest/failure/arrhythmia	☐	☐	☐	☐	☐	☐	☐	☐
3. Compartment syndrome	☐	☐	☐	☐	☐	☐	☐	☐
4. Cutaneous injury (e.g., pressure sore)	☐	☐	☐	☐	☐	☐	☐	☐
5. Delirium/altered mental status	☐	☐	☐	☐	☐	☐	☐	☐
6. Dysphagia/dysphonia	☐	☐	☐	☐	☐	☐	☐	☐
7. Fall	☐	☐	☐	☐	☐	☐	☐	☐
8. GI bleed	☐	☐	☐	☐	☐	☐	☐	☐
9. Hematoma	☐	☐	☐	☐	☐	☐	☐	☐
10.Ileus/bowel obstruction	☐	☐	☐	☐	☐	☐	☐	☐
11. Implant/instrumentation related:								
a. Loss of reduction/alignment/correction	☐	☐	☐	☐	☐	☐	☐	☐
b. Peri-implant fracture	☐	☐	☐	☐	☐	☐	☐	☐
c. Joint instability/dislocation	☐	☐	☐	☐	☐	☐	☐	☐
d. Aseptic loosening	☐	☐	☐	☐	☐	☐	☐	☐
12. Infection:								
a. Superficial wound	☐	☐	☐	☐	☐	☐	☐	☐
b. Deep wound	☐	☐	☐	☐	☐	☐	☐	☐
c. Urinary tract	☐	☐	☐	☐	☐	☐	☐	☐
d. Systemic	☐	☐	☐	☐	☐	☐	☐	☐
13. Myocardial infarction	☐	☐	☐	☐	☐	☐	☐	☐

Date (dd/mmm/yyyy): ___________________ Participant ID: _________________________

PAO ± Hip Arthroscopy Study

Post-op Adverse Events (Continued)								
14. Neurological deterioration:								
a. Sciatic nerve	☐	☐	☐	☐	☐	☐	☐	☐
b. Femoral nerve	☐	☐	☐	☐	☐	☐	☐	☐
c. Obturator nerve	☐	☐	☐	☐	☐	☐	☐	☐
d. Lateral femoral cutaneous nerve	☐	☐	☐	☐	☐	☐	☐	☐
f. Pudendal nerve	☐	☐	☐	☐	☐	☐	☐	☐
15. Non-union/mal union:								
a. Illium	☐	☐	☐	☐	☐	☐	☐	☐
b. Ischium	☐	☐	☐	☐	☐	☐	☐	☐
c. Pubis	☐	☐	☐	☐	☐	☐	☐	☐
16. Pain - new onset	☐	☐	☐	☐	☐	☐	☐	☐
17. Pneumonia	☐	☐	☐	☐	☐	☐	☐	☐
18. Renal insufficiency	☐	☐	☐	☐	☐	☐	☐	☐
19. Thrombolytic event:								
a. DVT	☐	☐	☐	☐	☐	☐	☐	☐
b. PE	☐	☐	☐	☐	☐	☐	☐	☐
20. Soft tissue reconstruction/repair fracture	☐	☐	☐	☐	☐	☐	☐	☐
21. Wound dehiscence	☐	☐	☐	☐	☐	☐	☐	☐
22. Wound drainage:								
a. Serous (requiring treatment)	☐	☐	☐	☐	☐	☐	☐	☐

24. How many days longer than expected did this patient stay in hospital due to adverse events (provide #): ___________ day(s)

25. Did complications require :

☐Not applicable

☐Revision

☐Reoperation

☐Readmisison

26. Treatment (select all that apply, and provide date of treatment) :

☐Arthroscopy Date:____

☐Hardware removal Date:____

☐Bone excision Date:____

☐Hip replacement Date:____

☐Wound I&D Date:____

☐Other Specify:____

Date:____

27. Other notes:
