# Supplementary material for: Periacetabular osteotomy with or without arthroscopic management in patients with hip dysplasia: study protocol for a multicenter randomized controlled trial
Source: Trials. 2020 Aug 18;21:725. doi: 10.1186/s13063-020-04592-9 (PMC7433104; doi:10.1186/s13063-020-04592-9)
Supplement: Supplementary file 5 — Additional file 5. International Hip Outcome Tool (iHOT-33). [file 13063_2020_4592_MOESM5_ESM.pdf]

Appendix E

APPENDIX 1

International Hip Outcome Tool (iHOT-33).

iHOT33

INTERNATIONAL HIP OUTCOME TOOL

NAME

DATE OF BIRTH

TODAY'S DATE

WHICH HIP IS THIS SURVEY ABOUT?

If we've asked you to tell us about one hip in particular, tick that. Otherwise, tick the one which causes most trouble.

◯ Left

◯ Right

QUALITY OF LIFE QUESTIONNAIRE FOR YOUNG, ACTIVE PEOPLE WITH HIP PROBLEMS

INSTRUCTIONS

- These questions ask about the problems you may be experiencing in your hip, how these problems affect your life, and the emotions you may feel because of these problems.
- Please indicate the severity by marking the line below each question with a slash.
  - » If you put a mark on the far **left**, it means that you **feel you are significantly impaired**. For example:

    SIGNIFICANTLY IMPAIRED /________________________________ NO PROBLEMS AT ALL

  - » If you put a mark on the far **right**, it means that you **do not think that you have any problems** with your hip. For example:

    SIGNIFICANTLY IMPAIRED ________________________________/ NO PROBLEMS AT ALL

  - » If the mark is placed in the middle of the line, this indicates that you are moderately disabled, or in other words, between the extremes of 'significantly impaired' and 'no problems at all'. It is important to put your mark at either end of the line if the extreme descriptions accurately reflect your situation.

TIP If you don't do an activity, imagine how your hip would feel if you had to try it.

- Please let your answers describe the typical situation in the last **month**.

SECTION 1 | SYMPTOMS AND FUNCTIONAL LIMITATIONS

The following questions ask about symptoms that you may experience in your hip and about the function of your hip with respect to daily activities. Please think about how you have felt most of the time over the past month and answer accordingly.

Q01 How often does your hip/groin ache?

CONSTANTLY ________________________________ NEVER

Q02 How stiff is your hip as a result of sitting/resting during the day?

EXTREMELY STIFF ________________________________ NOT STIFF AT ALL

Q03 How difficult is it for you to walk long distances?

EXTREMELY DIFFICULT ______ NOT DIFFICULT AT ALL

Q04 How much pain do you have in your hip while sitting?

EXTREME PAIN ______ NO PAIN AT ALL

Q05 How much trouble do you have standing on your feet for long periods of time?

SEVERE TROUBLE ______ NO TROUBLE AT ALL

Q06 How difficult is it for you to get up and down off the floor/ground?

EXTREMELY DIFFICULT ______ NOT DIFFICULT AT ALL

Q07 How difficult is it for you to walk on uneven surfaces?

EXTREMELY DIFFICULT ______ NOT DIFFICULT AT ALL

Q08 How difficult is it for you to lie on your affected hip side?

EXTREMELY DIFFICULT ______ NOT DIFFICULT AT ALL

Q09 How much trouble do you have with stepping over obstacles?

SEVERE TROUBLE ______ NO TROUBLE AT ALL

Q10 How much trouble do you have with climbing up/down stairs?

SEVERE TROUBLE ______ NO TROUBLE AT ALL

Q11 How much trouble do you have with rising from a sitting position?

SEVERE TROUBLE ______ NO TROUBLE AT ALL

Q12 How much discomfort do you have with taking long strides?

EXTREME DISCOMFORT ______ NO DISCOMFORT AT ALL

Q13 How much difficulty do you have with getting into and/or out of a car?

EXTREME DIFFICULTY ________________________________ NO DIFFICULTY AT ALL

Q14 How much trouble do you have with grinding, catching or clicking in your hip?

SEVERE TROUBLE ________________________________ NO TROUBLE AT ALL

Q15 How much difficulty do you have with putting on/taking off socks, stockings or shoes?

EXTREME DIFFICULTY ________________________________ NO DIFFICULTY AT ALL

Q16 Overall, how much pain do you have in your hip/groin?

EXTREME PAIN ________________________________ NO PAIN AT ALL

SECTION 2 | SPORTS AND RECREATIONAL ACTIVITIES

The following questions ask about your **hip** when you participate in sports and recreational activities. Please think about how you have felt most of the time over the past **month** and answer accordingly.

Q17 How concerned are you about your ability to maintain your desired fitness level?

EXTREMELY CONCERNED ________________________________ NOT CONCERNED AT ALL

Q18 How much pain do you experience in your hip after activity?

EXTREME PAIN ________________________________ NO PAIN AT ALL

Q19 How concerned are you that the pain in your hip will increase if you participate in sports or recreational activities?

EXTREMELY CONCERNED ________________________________ NOT CONCERNED AT ALL

Q20 How much has your quality of life deteriorated because you cannot participate in sport/recreational activities?

EXTREMELY DETERIORATED ________________________________ NOT DETERIORATED AT ALL

Q21 How concerned are you about cutting/changing directions during your sport or recreational activities?

☐ I do not do this action in my activities

EXTREMELY CONCERNED ______________________________ NOT CONCERNED AT ALL

Q22 How much has your performance level decreased in your sport or recreational activities?

EXTREMELY DECREASED ______________________________ NOT DECREASED AT ALL

SECTION 3 | JOB RELATED CONCERNS

The following questions relate to your hip with respect to your current work. Please think about how you have felt most of the time over the past month and answer accordingly.

☐ I do not work because of my hip *(please skip section)*
☐ I do not work for reasons other than my hip *(please skip section)*

Q23 How much trouble do you have pushing, pulling, lifting or carrying heavy objects at work?

☐ I do not do these actions in my activities

SEVERE TROUBLE ______________________________ NO TROUBLE AT ALL

Q24 How much trouble do you have with crouching/squatting?

SEVERE TROUBLE ______________________________ NO TROUBLE AT ALL

Q25 How concerned are you that your job will make your hip worse?

EXTREMELY CONCERNED ______________________________ NOT CONCERNED AT ALL

Q26 How much difficulty do you have at work because of reduced hip mobility?

EXTREME DIFFICULTY ______________________________ NO DIFFICULTY AT ALL

SECTION 4 | SOCIAL, EMOTIONAL AND LIFESTYLE CONCERNS

The following questions ask about social, emotional and lifestyle concerns that you may feel with respect to your hip problem. Please think about how you have felt most of the time over the past month and answer accordingly.

Q27 How frustrated are you because of your hip problem?

EXTREMELY FRUSTRATED ______________________________ NOT FRUSTRATED AT ALL

Q28 How much trouble do you have with sexual activity because of your hip?

☐ This is not relevant to me

SEVERE TROUBLE ______________________________ NO TROUBLE AT ALL

Q29 How much of a distraction is your hip problem?

EXTREME DISTRACTION ______________________________ NO DISTRACTION AT ALL

Q30 How difficult is it for you to release tension and stress because of your hip problem?

EXTREMELY DIFFICULT ______________________________ NOT DIFFICULT AT ALL

Q31 How discouraged are you because of your hip problem?

EXTREMELY DISCOURAGED ______________________________ NOT DISCOURAGED AT ALL

Q32 How concerned are you about picking up or carrying children because of your hip?

☐ I do not do this action in my activities

EXTREMELY CONCERNED ______________________________ NOT CONCERNED AT ALL

Q33 How much of the time are you aware of the disability in your hip?

CONSTANTLY AWARE ______________________________ NOT AWARE AT ALL
