# Supplementary material for: Periacetabular osteotomy with or without arthroscopic management in patients with hip dysplasia: study protocol for a multicenter randomized controlled trial
Source: Trials. 2020 Aug 18;21:725. doi: 10.1186/s13063-020-04592-9 (PMC7433104; doi:10.1186/s13063-020-04592-9)
Supplement: Supplementary file 6 — Additional file 6. PROMIS Global Health 10. [file 13063_2020_4592_MOESM6_ESM.pdf]

Appendix F

PROMIS Global Health 10

Date:________________________ Participant ID:________________ Time interval:_________________

Please respond to each item by marking one box per question

	Excellent	Very good	Good	Fair	Poor
In general, would you say your health is:	☐	☐	☐	☐	☐
In general, would you say your quality of life is:	☐	☐	☐	☐	☐
In general, how would you rate your physical health?	☐	☐	☐	☐	☐
In general, how would you rate your mental health, including your mood and your ability to think?	☐	☐	☐	☐	☐
In general, how would you rate your satisfaction with your social activities and relationships?	☐	☐	☐	☐	☐
In general, please rate how well you carry out your usual social activities and roles. (This includes activities at home, at work and in your community, and responsibilities as a parent, child, spouse, employee, friend, etc.)	☐	☐	☐	☐	☐

	Completely	Mostly	Moderately	A Little	Not At All
To what extent are you able to carry out your everyday physical activities such as walking, climbing stairs, carrying groceries, or moving a chair?	☐	☐	☐	☐	☐

**In the past 7 days**	Never	Rarely	Sometimes	Often	Always
How often have you been bothered by emotional problems such as feeling anxious, depressed or irritated?	☐	☐	☐	☐	☐

	None	Mild	Moderate	Severe	Very Severe
How would you rate your fatigue on average?	☐	☐	☐	☐	☐

How would you rate your pain on average?	☐	☐	☐	☐	☐	☐	☐	☐	☐	☐
	**No Pain**									**Worst Imaginable Pain**
