# Supplementary material for: Periacetabular osteotomy with or without arthroscopic management in patients with hip dysplasia: study protocol for a multicenter randomized controlled trial
Source: Trials. 2020 Aug 18;21:725. doi: 10.1186/s13063-020-04592-9 (PMC7433104; doi:10.1186/s13063-020-04592-9)
Supplement: Supplementary file 7 — Additional file 7. [file 13063_2020_4592_MOESM7_ESM.pdf]

Appendix G

Sink Classification (1)

**Grade**	**Description**
**I**	Not clinically relevant and required no deviation from routine postoperative care
**II**	Treated on outpatient basis or with close observation and resolved
**III**	Treatable and resolved with surgery or inpatient management
**IV**	Resulting in long-term deficit

Modes of Failure Classification(2)

**Mode**	**Description**
**1**	Progression of arthritis/organ failure resulting in joint replacement, leading to better clinical assessment of the state of disease in the hip joint using age as a possible surrogate and/or biomarker
**2**	Incorrect initial diagnosis/procedure, resulting in a different surgical intervention at re-operation, leading the clinician to advance their knowledge, and the scientific community to improve diagnostic accuracy
**3**	Malcorrection of the femur (Type A), the acetabulum (Type B), and the labrum (Type C), resulting in the same intervention being repeated, leading the clinician to analyze critically their surgical technique at re-operation, leading the clinicians to advance their knowledge and the scientific community to improve diagnostic accuracy
**4**	An unintended consequence of the initial surgical intervention such as adhesions, partial osteonecrosis of the femoral head or hip instability leading to further intervention.

References

1. Sink EL, Beaule PE, Sucato D, Kim YJ, Millis MB, Dayton M, Trousdale RT, Sierra RJ, Zaltz I, Schoenecker P, Monreal A, Clohisy J. Multicenter study of complications following surgical dislocation of the hip. J Bone Joint Surg Am. 2011;93(12):1132-6. Epub 2011/05/17. doi: 10.2106/jbjs.j.00794. PubMed PMID: 21571987.
2. Beaulé PE, Bleeker H, Singh A, Dobransky J. Defining modes of failure after joint-preserving surgery of the hip. The Bone and Joint Journal. 2017;99. doi: 10.1302/0301-620X.99B3.
