# Supplementary material for: Periacetabular osteotomy with or without arthroscopic management in patients with hip dysplasia: study protocol for a multicenter randomized controlled trial
Source: Trials. 2020 Aug 18;21:725. doi: 10.1186/s13063-020-04592-9 (PMC7433104; doi:10.1186/s13063-020-04592-9)
Supplement: Supplementary file 8 — Additional file 8: Figure 4. Schedule of enrolment, interventions, and assessments. [file 13063_2020_4592_MOESM8_ESM.pdf]

Appendix H

Figure 4. Schedule of enrolment, interventions, and assessments

| | Study Period | | | | | | |
|---|---|---|---|---|---|---|---|
| | **Enrolment** | **Allocation** | **Post-Allocation (Post-Surgery)** | | | | |
| **Timepoint** | **Pre Surgery** | | **Surgery** | **90-day** | **6 Months** | **12 Months** | **24 Months** |
| **Enrolment** | X | | | | | | |
| **Eligibiligy screen** | X | | | | | | |
| **Informed Consent** | X | | | | | | |
| **Allocation** | | X | | | | | |
| **Interventions** | | | | | | | |
| **PAO with or without scope** | | | X | | | | |
| **Assessments** | | | | | | | |
| Patient reported quality of life (iHOT-33) | X | | | | X | X | X |
| Performance Measures (Four Square Step Test and Sit-to-Stand five times test) | X | | | | X | X | X |
| Health related quality of life (PROMIS Global 10) | X | | | | X | X | X |
| Cartilage Quality (T1rho MRI) | X | | | | | | X |
| Adverse Events (Sink Classification System) | | | | X | | | |
| Reoperation/Failure Mode (Standardized failure mode) | | | | | | X | X |
| Work productivity (WPAI: SHP) | | | | | X | X | X |
