# Supplementary material for: Periacetabular osteotomy with or without arthroscopic management in patients with hip dysplasia: study protocol for a multicenter randomized controlled trial
Source: Trials. 2020 Aug 18;21:725. doi: 10.1186/s13063-020-04592-9 (PMC7433104; doi:10.1186/s13063-020-04592-9)
Supplement: Supplementary file 9 — Additional file 9. Work Productivity and Activity Impairment Questionnaire: Specific Health Problem V2.0 (WPAI:SHP). [file 13063_2020_4592_MOESM9_ESM.pdf]

Appendix I

Work Productivity and Activity Impairment Questionnaire:
Specific Health Problem V2.0 (WPAI:SHP)

The following questions ask about the effect of your PROBLEM on your ability to work and perform regular activities. *Please fill in the blanks or circle a number, as indicated.*

1. Are you currently employed (working for pay)? _____ NO ___ YES
   If NO, check "NO" and skip to question 6.

The next questions are about the **past seven days**, not including today.

2. During the past seven days, how many hours did you miss from work because of problems associated with your PROBLEM? *Include hours you missed on sick days, times you went in late, left early, etc., because of your PROBLEM. Do not include time you missed to participate in this study.*

   _____ HOURS

3. During the past seven days, how many hours did you miss from work because of any other reason, such as vacation, holidays, time off to participate in this study?

   _____HOURS

4. During the past seven days, how many hours did you actually work?

   _____HOURS *(If "0", skip to question 6.)*

5. During the past seven days, how much did your PROBLEM affect your productivity <u>while you were working</u>?

Think about days you were limited in the amount or kind of work you could do, days you accomplished less than you would like, or days you could not do your work as carefully as usual. If PROBLEM affected your work only a little, choose a low number. Choose a high number if PROBLEM affected your work a great deal.

Consider only how much PROBLEM affected productivity while you were working.

PROBLEM had no effect on my work	0	1	2	3	4	5	6	7	8	9	10	PROBLEM completely prevented me from working

CIRCLE A NUMBER

6. During the past seven days, how much did your PROBLEM affect your ability to do your regular daily activities, other than work at a job?

By regular activities, we mean the usual activities you do, such as work around the house, shopping, childcare, exercising, studying, etc. Think about times you were limited in the amount or kind of activities you could do and times you accomplished less than you would like. If PROBLEM affected your activities only a little, choose a low number. Choose a high number if PROBLEM affected your activities a great deal.

Consider only how much PROBLEM affected your ability to do your regular daily activities, other than work at a job.

PROBLEM had no effect on my daily activities	0	1	2	3	4	5	6	7	8	9	10	PROBLEM completely prevented me from doing my daily activities

CIRCLE A NUMBER
