# Supplementary material for: Periacetabular osteotomy with or without arthroscopic management in patients with hip dysplasia: study protocol for a multicenter randomized controlled trial
Source: Trials. 2020 Aug 18;21:725. doi: 10.1186/s13063-020-04592-9 (PMC7433104; doi:10.1186/s13063-020-04592-9)
Supplement: Supplementary file 10 — Additional file 10: Table 2. Study Gantt Chart. [file 13063_2020_4592_MOESM10_ESM.pdf]

Appendix J

Table 2. Study Gantt Chart

	**Year 1**		**Year 2**		**Year 3**		**Year 4**		**Year 5**	
**Event**	Q1/Q2	Q3/Q4	Q1/Q2	Q3/Q4	Q1/Q2	Q3/Q4	Q1/Q2	Q3/Q4	Q1/Q2	Q3/Q4
**Research Ethics Board**										
**Recruitment and Consent**										
**Patient Follow-up**										
**Cost Utility Analysis**										
**Interim & Final Data Analysis**										
**Knowledge Translation**										
