# Supplementary material for: Periacetabular osteotomy with or without arthroscopic management in patients with hip dysplasia: study protocol for a multicenter randomized controlled trial
Source: Trials. 2020 Aug 18;21:725. doi: 10.1186/s13063-020-04592-9 (PMC7433104; doi:10.1186/s13063-020-04592-9)
Supplement: Supplementary file 3 — Additional file 3. Exercise Diary. [file 13063_2020_4592_MOESM3_ESM.pdf]

Appendix C
Exercise Diary

Instructions for study participant: Please complete 3 sets of 10 repetitions for each exercise and write the date you completed the exercise. Exercises must be done every other day:

		Week 1 (post op week 5)				Week 2 (post op week 6)			
		Date	Date	Date	Date	Date	Date	Date	Date
Standing open-chain active hip abduction and flexion	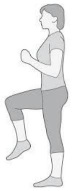								
Knee extension sitting	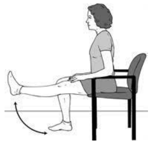								
		Week 3 (post op week 7)				Week 4 (post op week 8)			
		Date	Date	Date	Date	Date	Date	Date	Date
Step up/step down	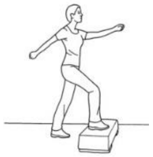								
Side Stepping (with resistance band)	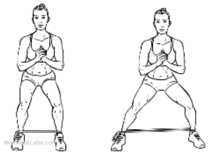								
